# Analysis of *Triticum aestivum* seedling response to the excess of zinc

**DOI:** 10.1007/s00709-015-0816-3

**Published:** 2015-04-24

**Authors:** Sława Glińska, Magdalena Gapińska, Sylwia Michlewska, Elżbieta Skiba, Jakub Kubicki

**Affiliations:** Laboratory of Microscopy Imaging and Advanced Biological Technics, Faculty of Biology and Environmental Protection, University of Lodz, Banacha 12/16, 90-237 Lodz, Poland; Institute of General and Ecological Chemistry, Lodz University of Technology, Żeromskiego 116, 90-924 Lodz, Poland

**Keywords:** Zinc toxicity, Wheat, Growth parameters, Mitotic index, Microelement content, Ultrastructure

## Abstract

The effects of 50 and 300 mg L^−1^ Zn^2+^ (50 Zn and 300 Zn) were investigated in *Triticum aestivum* (cv. Żura) grown hydroponically for 7 days. Although wheat treated with 50 Zn took up relatively high amount of the metal (8,943 and 1,503 mg kg^−1^ DW in roots and shoots, respectively), none of the morphological and cytological parameters were changed. After 300 Zn, the metal concentration increased to 32,205 and 5,553 mg kg^−1^ DW in roots and shoots, respectively. It was connected with the depletion of shoot and root growth, their fresh and dry weight, water content and mitotic index of root meristematic cells. Microelement contents (Cu, Mn and Fe) after 50 Zn were changed only in roots, while 300 Zn disturbed ion balance in whole plants. The most evident ultrastructural alterations of root meristematic cells caused by both tested Zn^2+^ doses included increased vacuolization, accumulation of granular deposits inside vacuoles and cell wall thickening. The effect of 300 Zn on root cell ultrastructure was greater that of 50 Zn. The majority of mitochondria had condensed matrix and swollen cristae, plastids contained plastoglobuli, nucleoli were ring-shaped, thinned down cytoplasm with lipid droplets and swollen endoplasmic reticulum cisternae appeared. In mesophyll cells, 50 Zn caused slight reorganization of chloroplast thylakoids and formation of condensed mitochondria. Three hundred Zn triggered more extensive, but not degenerative, changes: plasmolysis of some cells; chloroplasts with protrusions, changed thylakoid organisation and often large starch grains; irregular, condensed mitochondria. The results indicate that *T. aestivum* cv. Żura is relatively tolerant to Zn stress.

## Introduction

Zinc (Zn) is an essential element for all organisms because it is a structural constituent and regulatory co-factor in enzymes involved in many biochemical pathways (Broadley et al. 2007). Zn deficiency is currently listed as a high-risk factor for human health (Broadley et al. 2007). It occurs predominantly in the regions where soils are poor in available Zn, and cereals cultivated there are the major source of calorie intake (Alloway 2008). It is, therefore, highly important to develop quick solutions to the Zn-deficiency problem and increase Zn concentration in crops. The dietary Zn intakes can be improved through biofortification of edible crops. Wheat is the staple food crop for 35 % of the world’s population and provides more calories and protein in total than any other crop. The application of Zn-containing fertilizers increases the concentration of Zn in wheat grain up to threefold (Cakmak 2009).

Zinc, together with other plant micronutrients, can limit growth when it is present both at low and at excessive concentrations due to deficiency and toxicity, respectively. High concentrations of available zinc in soils usually arise from various sources of pollution, including atmospheric deposition from a nearby industrial source, such as smelting works, flooding of alluvial soils with zinc-polluted water and sediments, excessive applications of zinc rich materials, including pig and poultry manures from animals fed additional zinc, and high zinc sewage sludge or industrial waste waters (Alloway 2008; Tsonev and Lidon 2012). Zn concentration in municipal sewage sludge used as organic soil fertilizers can reach 1,000 mg kg^−1^ (Gondek et al. 2012). Although the accumulation of zinc to potentially toxic levels in crops is a small probability, it is important to constantly monitor the ion level in fertilized soil and in plants growing on it.

The Institute of Soil Science and Plant Cultivation (IUNG) determined threshold values of Zn concentration in agricultural light soils from 50 mg kg^−1^ of dry soil matter in soils of class 0 (natural content) to 300 mg kg^−1^ in soils of class II (slightly contaminated soils that could be used for agriculture; Szczepocka 2005).

Zn toxicity symptoms in plants usually occur when Zn leaf concentration is above 300 mg kg^−1^ DW, although toxicity thresholds can be highly variable even within the same species (Broadley et al. 2007). There are species that tolerate significantly higher Zn levels in their tissues. The concentration of Zn in plants growing in the polluted area near the Biała river in Silesian Voivodeship in Poland highly exceeded that value without serious symptoms of toxicity, e.g. *Mentha aquatica* leaves 576 mg kg^−1^ DW and *Carex* sp. roots 6,500 mg kg^−1^ DW (Szczepocka 2005).

Within the same species, significant differences may occur as far as Zn sensitivity is concern. A Zn-hyperaccumulating ecotype of *Sedum alfredi* grew well without any obvious change when exposed to Zn up to 500 μM, while toxic effects such as extremely stunted roots, thickened cuticule and cracked, brown and wilted leaves were observed in a non-Zn-hyperaccumulating ecotype of this species exposed to 50 μM Zn (Jin et al. 2008).

The aim of this study was to determine the morphological, cytological and ultrastructural effects of Zn^2+^ at the concentrations of 50 and 300 mg L^−1^ on *Triticum aestivum* (cv. Żura), an important crop plant, growing hydroponically under controlled laboratory conditions.

## Material and methods

### Plant material and treatment

Seeds of *T. aestivum* (cv. Żura) were surface sterilized with 70 % ethanol for 10 min, and then rinsed extensively with distilled water. Subsequently, they were placed on moistened filter paper in petri dishes to germinate in the darkness at 22 °C for 2 days. Then seedlings were hydroponically grown for 7 days on aerated Hoagland solution containing: KNO_3_ (0.51 g L^−1^), Ca(NO_3_)_2_×4H_2_O (1.18 g L^−1^), MgSO_4_×7H_2_O (0.49 g L^−1^), KH_2_PO_4_ (0.14 g L^−1^), H_3_BO_3_ (0.6 mg L^−1^), MnCl_2_×4H_2_O (0.4 mg L^−1^), ZnSO_4_×7H_2_O (0.05 mg L^−1^), CuSO_4_×5H_2_O (0.05 mg L^−1^), FeEDTA (10.28 mg L^−1^) and Na_2_MoO_4_×2H_2_O (0.2 mg L^−1^) at pH 6.5 and cultured at 21 °C. The plants were growing under controlled conditions: light intensity of 170 μE m^−2^ s^−1^, 16/8-h day/night photo-period and temperature 21 °C for 4 days. The growth medium was changed every 48 h. After that time, the material was treated with 400 mL of the medium supplemented with Zn^2+^ to the concentrations of 50 and 300 mg L^−1^ (25 plants each). Plants cultured in standard Hoagland solution were the control. The solutions were changed every 24 h. The experiment was repeated six times.

### Growth parameters

Root and shoot growth was determined after 1, 3 and 7 days of incubation by subtracting the length of roots/shoots before incubation from that after incubation. Shoot and root fresh (FW) and dry weight (DW) were evaluated at the end of the experiment (7th day). To estimate DW, the plant material was dried for 2 days at 60 °C.

### Water content

The water content (WC) was calculated from FW and DW values according to the equation:

$$ \left[\%\right]\ \mathrm{W}\mathrm{C} = \left(\mathrm{F}\mathrm{W} - \mathrm{D}\mathrm{W}\right) \times {\mathrm{FW}}^{-1} \times 100 $$

### Mitotic index

Mitotic index of root meristematic cells was determined after 1, 3 and 7 days of the experiment. Root meristems, 10 per each experimental series, after fixation in Carnoy’s solution, were stained with Schiff reagent and squashed specimens were made. Subsequently, mitotic index (MI) was calculated according to the equation:

$$ \left[\%\right]\ \mathrm{M}\mathrm{I} = \mathrm{the}\ \mathrm{number}\ \mathrm{of}\ \mathrm{dividing}\ \mathrm{cells}\ /\mathrm{the}\ \mathrm{number}\ \mathrm{of}\ \mathrm{scored}\ \mathrm{cells}\ \left(1000\ \mathrm{per}\ \mathrm{root}\right) \times 100 $$

### Microelement analysis

In order to determine contents of zinc and other essential microelements (Cu, Mn and Fe) in roots and shoots, 0.2 mg of dried plant material (washed in deionized water before drying) was digested with the mixture of 6 mL of concentrated HNO_3_ and l mL of 30 % HCl in a closed system at 240 °C in a microwave oven Multivawe 3000 (Anton Paar) for 50 min. The concentration of Zn was determined by Flame Atomic Absorption Spectrometry (FAAS) using (GBC 932 plus, Australia). Calibration was made using a multi-element standard (Merck).

### Transmission electron microscopy (TEM)

From the control and treated plants, small portions of five leaves were cut from their central parts and five root meristems were cut off from different plants. The lamina samples were fixed in 3 % glutaraldehyde in 0.1 M cacodylate buffer pH 6.8, for 3 h, and the roots in 2 % glutaraldehyde in 0.1 M cacodylate buffer pH 7.2, for 2 h, both at 4 °C. Subsequently, they were rinsed with the same buffer and post-fixed in osmium tetraoxide (the leaves in 2 % and the roots in 1 % solution) for 2 h at 4 °C. The material was dehydrated in a graded ethanol series and embedded in Epon-Spur’s resin mixture. The 80-nm-thick cross sections of the leaves and longitudinal sections of the roots were obtained with an ultramicrotome (Ultracut E, Reichert Yung, Germany). The ultrathin sections were placed on formvar-coated nickel grids and stained with a saturated solution of uranyl acetate, and subsequently, with lead citrate (Reynolds 1963). The cell ultrastructure was analysed on TEM (JEM 1010, JEOL, Japan) at 80 kV.

### Statistical analysis

The data are shown as means with the standard error (SE). The significance of differences between treatments was determined by the *t* Student test. Differences at *p* ≤ 0.05 were considered statistically significant.

## Results

### Growth parameters

**Fig. 1:**
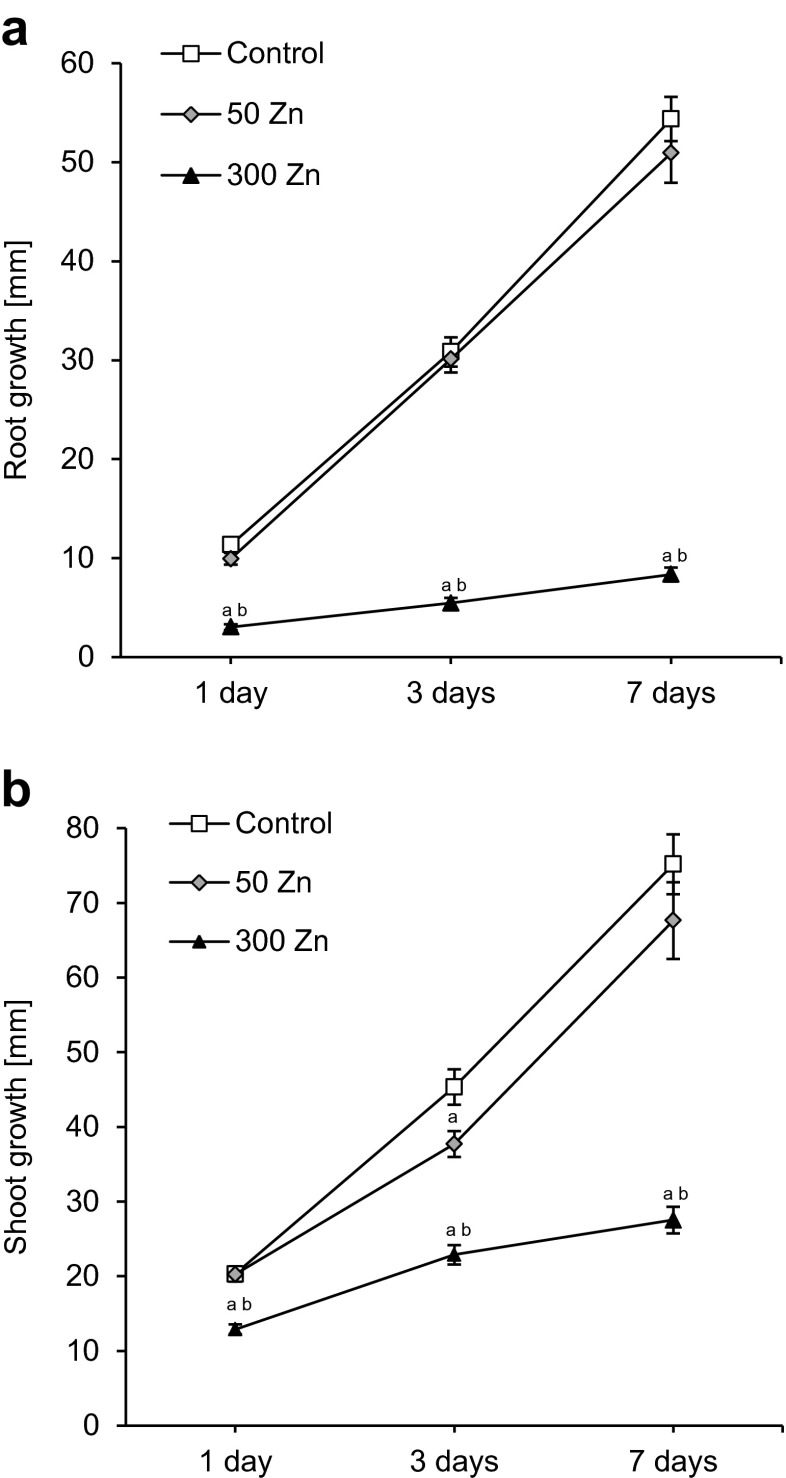
The effect of 50 and 300 mg L^−1^ Zn on root (**a**) and shoot (**b**) growth rate of *Triticum aestivum* plants during a 7-day treatment. Values are means of 60 measurements (6 replicates with 10 plants), and *vertical bars* represent SE. *a* denotes significant differences between Zn-treated plants and the control, and *b* between 50 and 300 Zn variants at *p* ≤ 0.05

Fifty Zn did not affect root growth during a 7-day treatment (Fig. 1a). After 1, 3 and 7 days of the experiment, the root length increased similarly as in the control material by approximately 10, 30 and 50 mm, respectively. The shoot growth decreased slightly (by 17 %) on the third day of experiment; but after longer exposure to 50 Zn, it did not differ significantly from the control material (Fig. 1b).

The root and shoot growth of *T. aestivum* was strongly impaired by 300 Zn (Fig. 1). The root growth fell dramatically after the 1st day of treatment (by 74 %), and the effect persisted causing 82 and 85 % decreases after 3rd and 7th day, respectively (Fig. 1a). The wheat shoots were slightly less sensitive to high Zn dose than roots; however, their growth was also reduced already after 1 day of experiment (by 36 %). The decrease in shoot growth after 3rd and 7th day of treatment amounted to 50 and 63 %, respectively (Fig. 1b).

**Fig. 2:**
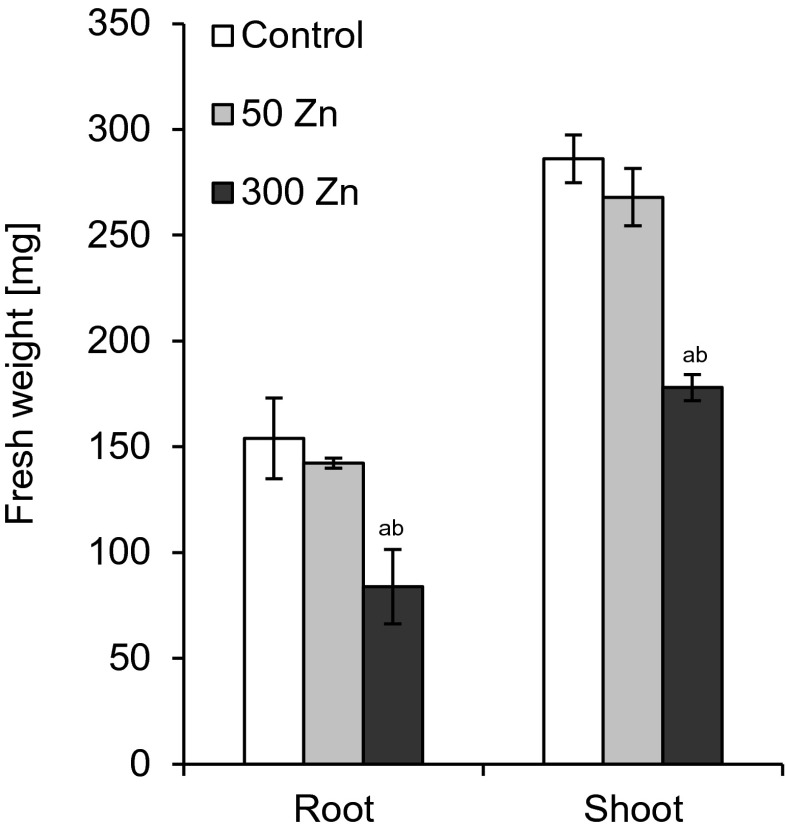
The effect of 50 and 300 mg L^−1^ Zn on root and shoot fresh weight of *Triticum aestivum* plants after a 7-day treatment. Values are means of six replicates, and *vertical bars* represent SE. *a* denotes significant differences between Zn-treated plants and the control, and *b* between 50 and 300 Zn variants at *p* ≤ 0.05

**Fig. 3:**
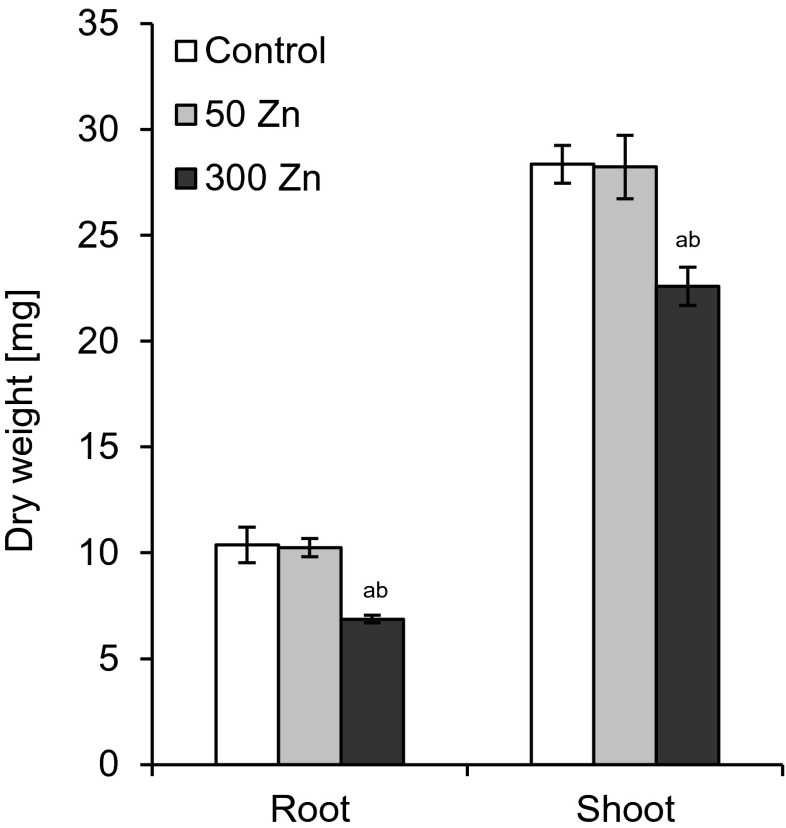
The effect of 50 and 300 mg L^−1^ Zn on root and shoot dry weight of *Triticum aestivum* plants after a 7-day treatment. Values are means of six replicates, and *vertical bars* represent SE. *a* denotes significant differences between Zn-treated plants and the control, and *b* between 50 and 300 Zn variants at *p* ≤ 0.05

Root and shoot biomass production was estimated after 7 days of experiment. The low Zn dose did not change these parameters as compared to the control (Figs. 2 and 3). FW and DW were reduced significantly following 300 Zn treatment (Figs. 2 and 3), with the roots being affected more than the shoots. The FW and DW of the former dropped to 54 and 66 % of the control while the respective values for the latter to 62 and 80 % (Figs. 2 and 3).

### Water content

**Fig. 4:**
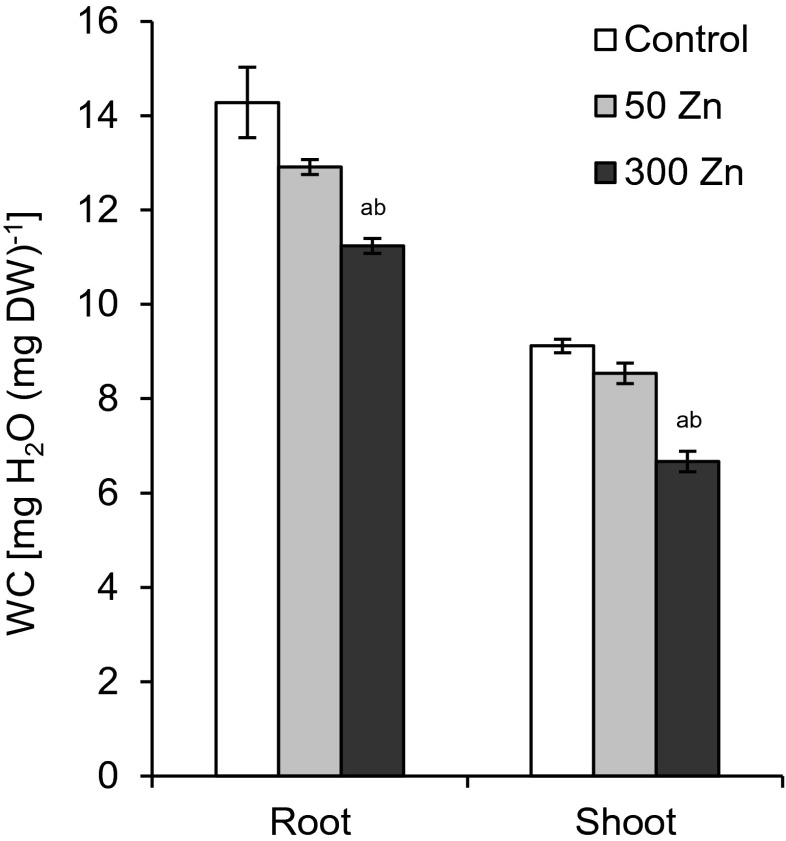
The effect of 50 and 300 mg L^−1^ Zn on root and shoot water content (WC) of *Triticum aestivum* plants after a 7-day treatment. Values are means of six replicates, and *vertical bars* represent SE. *a* denotes significant differences between Zn-treated plants and the control, and *b* between 50 and 300 Zn variants at *p* ≤ 0.05

WC in both analysed organs of *T. aestivum* was not changed by 50 Zn treatment; however, the higher Zn concentration diminished this parameter to 78 and to 74 % of the values noted in the control roots and shoots, respectively (Fig. 4).

### Mitotic index

**Fig. 5:**
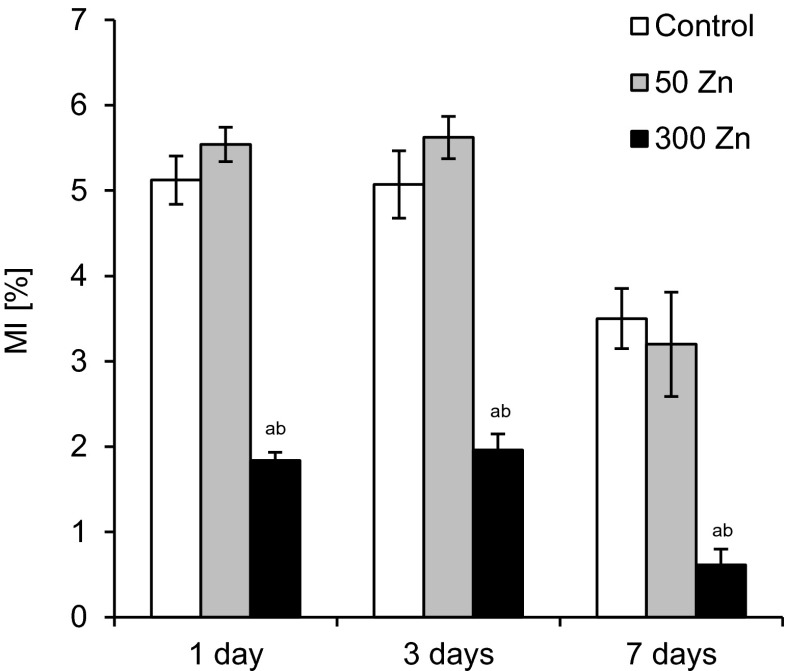
The effect of 50 and 300 mg L^−1^ Zn on mitotic index (MI) of root meristematic cells of *Triticum aestivum* plants during a 7-day treatment. Values are means of 10 replicates, and *vertical bars* represent SE. *a* denotes significant differences between Zn-treated plants and the control, and *b* between 50 and 300 Zn variants at *p* ≤ 0.05

Similarly as in the case of root growth, treatment with the lower dose of Zn did not significantly affect MI in root-tip cells throughout the experiment, while 300 Zn reduced the value of this parameter by 64, 61 and 83 %, after 1, 3 and 7 days, respectively, as compared to the control (Fig. 5).

### Microelement analysis

The contents of Zn in *T. aestivum* cultivated under the control conditions were 145.6 and 60.6 mg kg^−1^ DW in roots and shoots, respectively (Table 1). Also other analysed microelements (Cu, Mn and Fe) were more abundant in the roots (Table 1).

**Table 1:** The effect of 50 and 300 mg L^−1^ Zn^2+^ on microelement content in shoot and root of *Triticum aestivum* plants after a 7-day treatment

		Microelement content [mg kg^−1^ DW]			
		Zn	Cu	Mn	Fe
Control	Shoot	60.6 ± 27.2	9.7 ± 3.2	15.6 ± 5.5	30.0 ± 5.5
	Root	145.6 ± 43.2	27.0 ± 11.8	68.5 ± 11.2	33.1 ± 2.0
50 Zn	Shoot	1,503.3 ± 178.4 a	6.1 ± 1.0	11.6 ± 4.9	27.9 ± 1.9
	Root	8,942.9 ± 547.1 a	73.4 ± 17.3 a	12.7 ± 6.6 a	53.3 ± 1.4 a
300 Zn	Shoot	5,553.1 ± 576.8 ab	3.5 ± 1.4 ab	9.5 ± 2.3 a	19.6 ± 4.7 ab
	Root	32,205.2 ± 8928.4 ab	109.0 ± 5.3 ab	4.3 ± 1.6 a	679.0 ± 97.8 ab

Each value represents a mean of 3 replicates ± SE. The *a* denote significant differences of Zn-treated plants from control and *b* between 50 and 300 Zn variants at *p* ≤ 0.05

Fifty Zn significantly enhanced this ion content, the 25-fold and 61-fold increases were observed in the shoots and roots, respectively. However, other microelement concentrations were changed in the roots, but not in the shoots (Table 1). Fifty Zn increased Cu and Fe absorption by the roots up to 272 and 161 % of the control, respectively. Mn ion content in the roots decreased to 19 % of the control value.

Three hundred Zn drastically raised the Zn content both in the roots (221-fold) and in the shoots (92-fold; Tab. 1). The other microelement concentrations were also affected both in the roots and the shoots (Table 1). In the shoots, Cu, Mn and Fe concentrations declined to 36, 61 and 65 % of the control, respectively. In the roots, Mn content drastically diminished to 6 % of the control while those of Cu and Fe significantly raised (4-fold and 20-fold, respectively).

### TEM

**Fig. 6:**
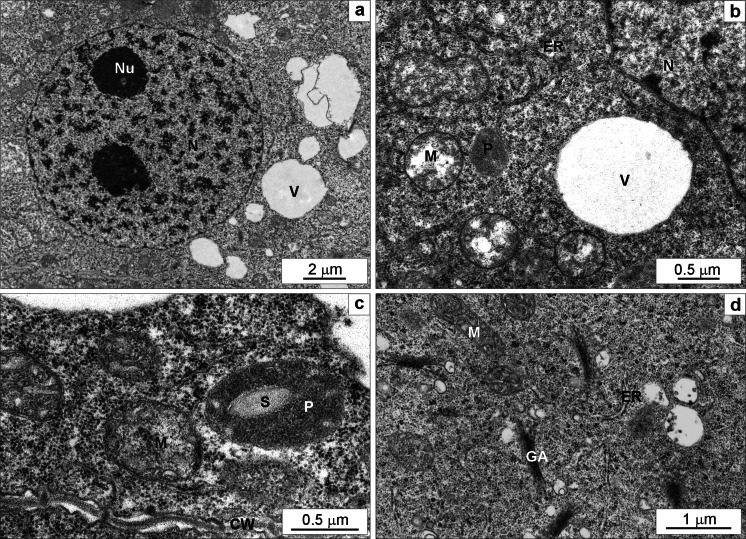
The ultrastructure of root meristematic cells of the control *Triticum aestivum* plants after a 7-day hydroponic cultivation. The part of a cell with typical nucleolus containing two nucleoli and small electron-transparent vacuoles scattered in the cytoplasm (**a**). Mitochondria with low-density matrix and narrow cristae, a small plastid without starch, endoplasmic reticulum cisternae running in different directions and an electron-lucent vacuole (**b**). Mitochondria with numerous slightly widened cristae, a plastid with a starch grain, endoplasmic reticulum cisternae and a cell wall with visible plasmodesmata (**c**). Numerous active Golgi apparati with quite numerous vesicles, mitochondria with numerous slightly widened cristae, endoplasmic reticulum cisternae running in different directions (**d**). *CW* cell wall, *ER* endoplasmic reticulum, *GA* Golgi apparatus, *M* mitochondrium, *N* nucleus, *Nu* nucleolus, *P* plastid, *S* starch, *V* vacuole

Ultrastructure of meristematic cells of the control wheat roots on the 7th day of the experiment was typical (Fig. 6). The central part of the cell was occupied by a nucleus with 1–3 nucleoli (Fig. 6a). The level of cell vacuolization was low and small vacuoles were electron-transparent (Fig. 6a, b). Plastids had electron-dense stroma and rare thylakoids, small starch grains were seen sporadically, and no plastoglobuli were visible (Fig. 6b, c). Mitochondria displayed electron-transparent matrix and usually narrow cristae (Fig. 6b–d). Single endoplasmic reticulum cisternae were running in different directions (Fig. 6b–d). Golgi apparatus was composed of 5–6 cisternae and a few vesicles (Fig. 6d). Cell wall was thin with visible plasmodesmata (Fig. 6c).

**Fig. 7:**
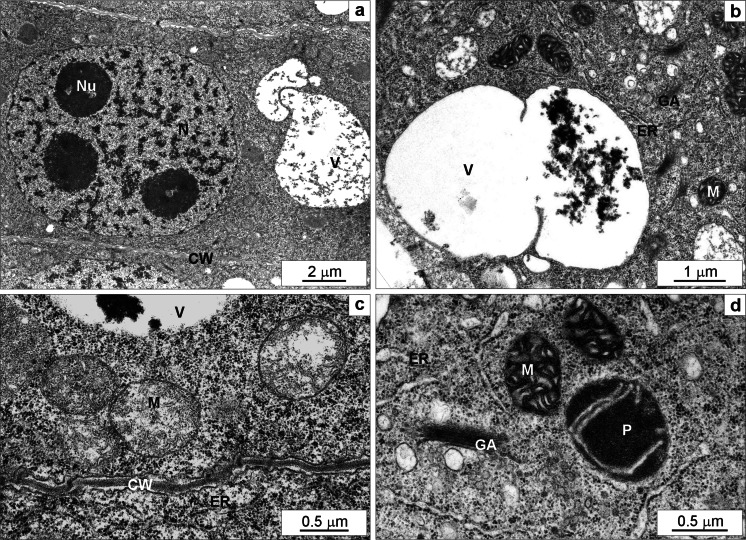
The ultrastructure of root meristematic cells of *Triticum aestivum* plants after a 7-day treatment with 50 mg L^−1^ Zn. The part of a cell with typical nucleolus containing three nucleoli, large vacuoles containing numerous small granular deposits and a typical thin cell wall (**a**). A large vacuole containing electron-dense material, mitochondria with condensed matrix and numerous wide cristae, endoplasmic reticulum and numerous Golgi apparati visible in the cytoplasm (**b**). Mitochondria with low-density matrix and narrow cristae, endoplasmic reticulum cisternae, thin cell wall and a fragment of a vacuole with electron-dense material (**c**). Active Golgi apparatus with quite large vesicles, condensed type of mitochondria with numerous widened cristae, slightly swollen endoplasmic reticulum cisternae, a plastid with electron-dense stroma and a few thylakoids (**d**). *CW* cell wall, *ER* endoplasmic reticulum, *GA* Golgi apparatus, *M* mitochondrium, *N* nucleus, *Nu* nucleolus, *P* plastid, *S* starch, *V* vacuole

Fifty Zn-enhanced cell vacuolization. The vacuoles were filled with countless small granules and frequently large electron-dense deposits occurred (Fig. 7a, b). There were many typical mitochondria (Fig. 7c), but sometimes they turned into a condensed form, *i.e.* with electron-dense matrix and numerous swollen cristae (Fig. 7b, d). Golgi apparatus cisternae with numerous vesicles around them were visible in the cytoplasm (Fig. 7b, d). Plastids had electron-dense stroma with few thylakoids (Fig. 7d). Cell wall was thicker and sometimes slightly wavy (Fig. 7c).

**Fig. 8:**
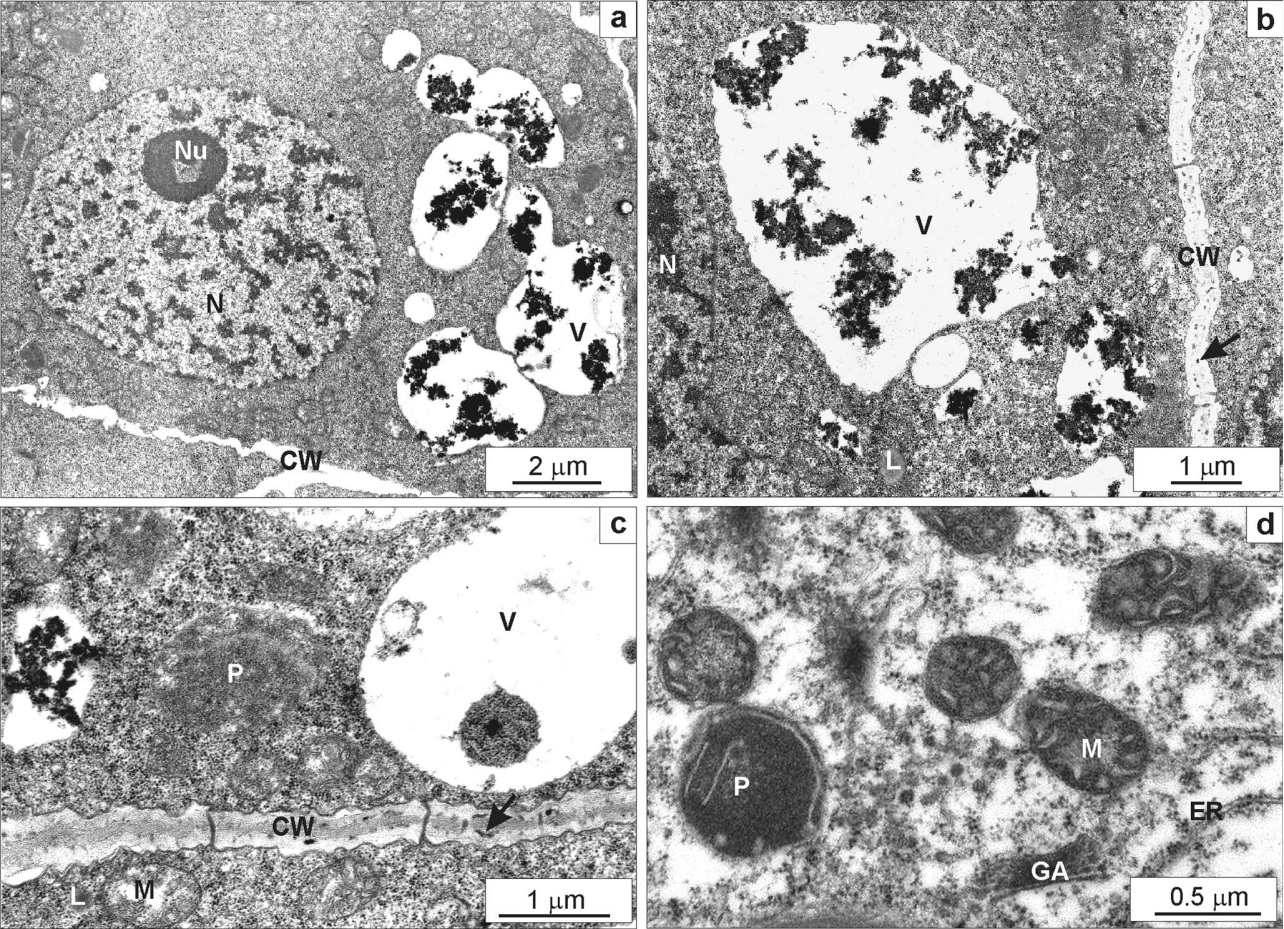
The ultrastructure of root meristematic cells of *Triticum aestivum* plants after a 7-day treatment with 300 mg L^−1^ Zn. A nucleus containing nucleolus with a vacuole, large vacuoles containing numerous large electron-dense deposits and a wavy cell wall (**a**). Large vacuoles containing electron-dense material, a lipid droplet visible in the cytoplasm and a cell wall containing small electron-dense deposits (*arrow*; **b**). Mitochondria with low-density matrix and narrow cristae, a plastid without starch, a lipid droplet in the cytoplasm, a cell wall with electron-dense deposits (*arrow*) and visible plasmodesmata, and fragments of vacuoles with electron-dense material (**c**). A fragment of a cell with thinned down cytoplasm, non-active Golgi apparatus without vesicles, condensed type of mitochondria, a plastid with electron-dense stroma and a few thylakoids and narrow endoplasmic reticulum cisternae (**d**). *CW* cell wall, *ER* endoplasmic reticulum, *GA* Golgi apparatus, *L* lipid, *M* mitochondrium, *N* nucleus, *Nu* nucleolus, *P* plastid, *V* vacuole

Three hundred Zn significantly affected root cell ultrastructure. It triggered far more extensive vacuolization than the lower Zn dose. After 7 days of treatment, large vacuoles were packed with numerous huge electron-dense deposits (Fig. 8a–c). Small granules were also seen in irregularly thickened cell wall (Fig. 8b, c). The majority of mitochondria were in condensed form (Fig. 8d). Plastids contained plastoglobuli (Fig. 8d). The cytoplasm was thinned down in some cells, and lipid droplets were seen in it (Fig. 8b–d). Sometimes swollen endoplasmic reticulum cisternae, and quite frequent ring-shaped nucleoli occurred (Fig. 8a).

**Fig. 9:**
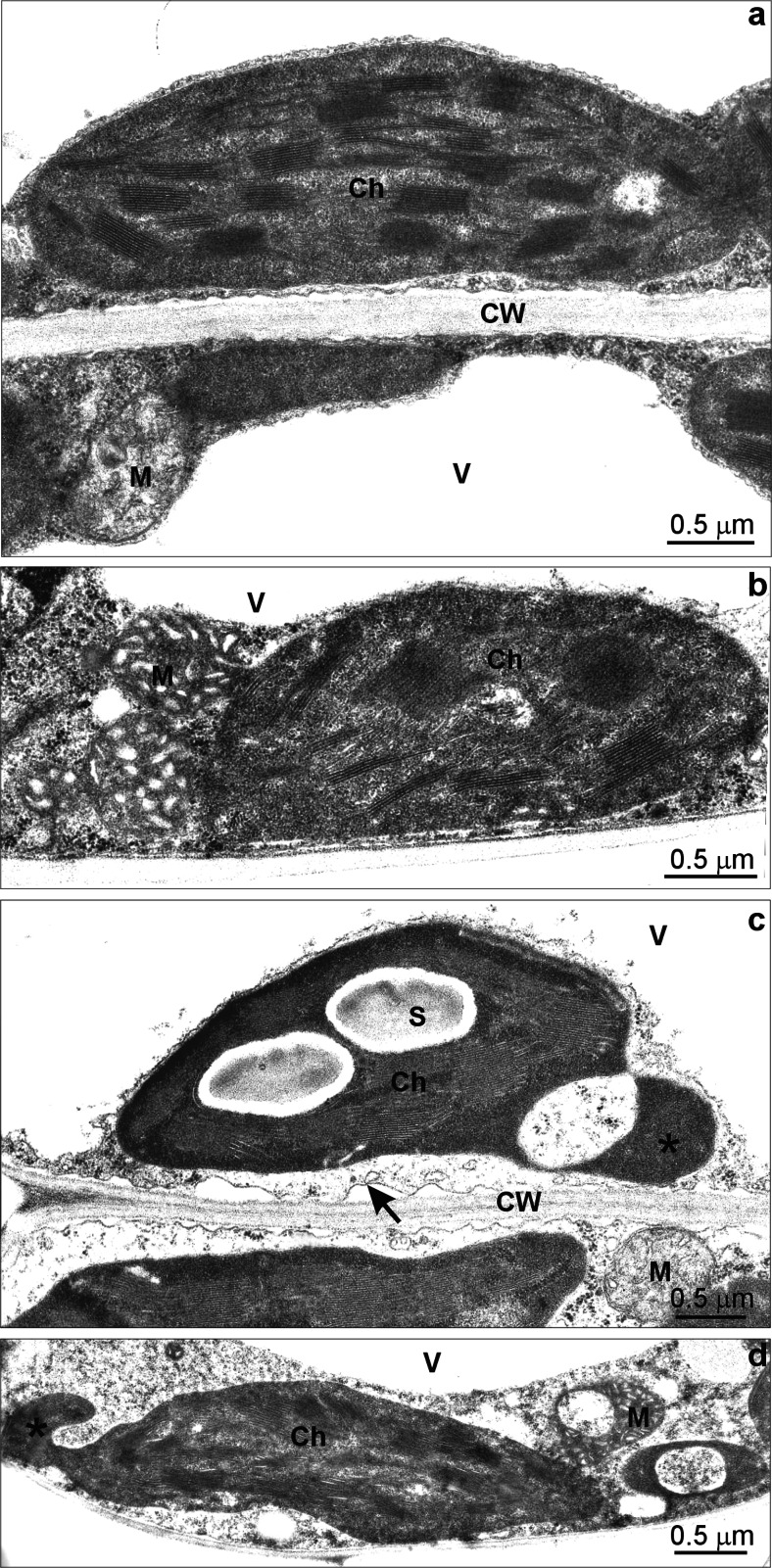
The ultrastructure of leaf mesophyll cells of *Triticum aestivum* plants after a 7-day treatment with 50 and 300 mg L^−1^ Zn. Fragment of a leaf mesophyll cell from the control material with a thin layer of cytoplasm surrounding the transparent vacuoles adhering to a typical cell wall. An ellipsoid chloroplast with regularly organized grana and a small starch grain, a mitochondrium with a matrix of electron density similar to cytoplasm with narrow cristae (**a**). A fragment of a mesophyll cell from 50 Zn-treated material containing a chloroplast with slightly changed organization of thylakoids, condensed type of mitochondria and a fragment of electron-transparent vacuole (**b**). A fragment of a mesophyll cell from 300 Zn-treated material with symptoms of plasmolysis (*arrow*) and slightly thinned down cytoplasm, an electron-transparent vacuole, a chloroplast with dense stroma, not regular thylakoid organisation and large starch grains forming protrusion (*asterisk*) and a mitochondrium with low-density matrix and narrow cristae (**c**). A fragment of a mesophyll cell from 300 Zn-treated materials with slightly thinned down cytoplasm, an electron-transparent vacuole, a chloroplast with disorganised thylakoid system and a protrusion (*asterisk*), changed in shape mitochondria with swollen cristae and condensed matrix (**d**). *Ch* chloroplast, *CW* cell wall, *M* mitochondrium, *S* starch, *V* vacuole

Ultrastructure of mesophyll cells of the control wheat leaves on 7th day of the experiment was typical with thin layer of cytoplasm surrounding the central transparent vacuole (Fig. 9a). Ellipsoid chloroplasts had regularly organized grana and starch grains were very rare and small. Mitochondria, oval in shape with matrix of electron-density similar to cytoplasm, had narrow cristae (Fig. 9a).

The mesophyll cells of the wheat leaves treated with 50 Zn did not differ significantly from the control. Sometimes the cells contained chloroplasts with slightly changed organization of thylakoids and mitochondria with swollen cristae and condensed matrix (Fig. 9b).

Three Zn triggered far more extensive changes in the leaf mesophyll cell ultrastructure. Some cells had slightly thinned down cytoplasm and showed symptoms of plasmolysis (Fig. 9c, arrow). The vacuoles did not contain any electron-dense deposits (Fig. 9c, d). Chloroplasts showed less regular shape, dense stroma and irregular thylakoid organisation (Fig. 9c). Moreover, specific protrusions containing thylakoid-free stroma were observed (Fig. 9c, d; asterisk). Many chloroplasts contained large starch grains (Fig. 9c). Two types of mitochondria were noticed: (i) similar to those in the control material (Fig. 9c) and (ii) changed in shape with swollen cristae and condensed matrix (Fig. 9d).

## Discussion

Growth parameters are good indicators of stress, and their changes were noticed after plant exposure to different heavy metals (Sagardoy et al. 2009; Jain et al. 2010; Yang et al. 2011; Caldelas et al. 2012). In our experiment, a decline of FW and DW of shoots and roots as well as of their growth was observed after application of 300 Zn. Among the analysed parameters, the root growth was the most responsive one as after 7 days of treatment it decreased by 85 % while FW and DW only by 46 and 34 %, respectively. Much smaller amount of Zn was accumulated in the shoots, this might be the reason why the toxic effect was less evident in them.

The decline in biomass production might be explained by the inhibition of meristematic cell division and of elongation of root cells. 300 Zn-triggered sharp depression in the MI of wheat root meristematic cells just after 1 day of Zn application. Similarly, 88 % inhibition of cell division in the presence of Zn at the concentration of 130 mg L^−1^ was observed in *Saccharum* spp (Jain et al. 2010).

The levels of Zn in plants usually range between 10 and 100 mg kg^−1^ of DW and toxicity symptoms usually become visible in crop species at Zn > 300 mg kg^−1^ leaf DW (Broadley et al. 2007). In our experiment, the wheat cultivated in Hoagland solution (control) had 60 mg Zn per kg of DW of shoot, and such an amount ensured optimal plant growth. Wheat has relatively high tolerance to Zn. The plants treated with 50 Zn contained 1,503 mg Zn kg^−1^ shoot DW and 8,942 mg Zn kg^−1^ root DW but no toxicity symptoms were observed. Similarly to wheat, *A. thaliana* seedlings containing 1980 mg Zn kg^−1^ shoot DW were characterised by normal growth and lack of morphological symptoms of Zn toxicity (Richard et al. 2011). *P. tomentosa* grew at a nutrient solution containing similar Zn dose (1,000 μM) was without any adverse effect but it absorbed a smaller amount of this microelement (about 1,000 mg kg^−1^ root DW and 400 mg kg^−1^ shoot DW) (Azzarello et al. 2012). Zn hyperaccumulator *Arabis paniculata* accumulated up to 10,000 mg Zn kg^−1^ shoot DW, and no biomass decrease was observed (Zeng et al. 2011). Wheat treated with 300 Zn accumulated 5553 mg Zn kg^−1^ shoot DW and as much as 32205 mg Zn kg^−1^ root DW but this dose was highly toxic reducing FW and DW as well as decreasing root and shoot growth. The significant reduction of growth parameters (by about 60 %) in *Saccharum* spp. was reported already at 130 mg L^−1^ Zn concentration (Jain et al. 2010). Other species are more sensitive to Zn excess. The significant decrease in the total DW and leaf area of *Paulownia tomentosa* grown hydroponically was observed already at 2,000 μM Zn (Azzarello et al. 2012), whereas *Pisum sativum* became inhibited after 1,000 μM Zn application (Doncheva et al. 2001).

Zn, Cu, Mn and Fe are the micronutrients which are essential for plants and their homeostasis seems to be very important (Palmer and Guerinot 2009). Supply of Zn into the growth medium affected Cu, Mn and Fe accumulation in plants. In the roots their levels were changed after application of both tested Zn concentrations but in the shoots microelement content (Cu, Mn and Fe) decrease was triggered only by 300 Zn. The alterations in mineral content caused by Zn excess are often more evident in roots than in shoots (Caldelas et al. 2012; Jain et al. 2010). While Mn concentration declined both in the shoots and roots of the wheat plants, Cu and Fe reduction in the shoots was accompanied by an increase in their concentration in the roots which suggests rather disturbances in their transport than depletion of their uptake. Similarly, in *Beta vulgaris* grown hydroponically with Zn up to 300 μM, Fe concentration decreased in shoots and increased in roots while Mn concentration declined in both plant organs (Sagardoy et al. 2009). Jain et al. (2010) after 65 and 130 mg L^−1^ Zn^2+^ supplementation observed decrease in Cu content in *Saccharum* spp.; but contrary to our results, it concerned whole plants, not only shoots. Moreover, they noted decrease in Fe and slight increase in Mn contents in roots without significant changes of these ions contents in leaves after the higher Zn dose (Jain et al. 2010).

Nearly 90 % of Fe in plants is localized in chloroplasts, where it is required for electron transport chain and synthesis of chlorophyll-determining proper course of photosynthesis and biomass production (Palmer and Guerinot 2009). The 45 % decrease in Fe content in *T. aestivum* shoots may be one of the most important reasons of biomass production decline.

Zn overdose altered not only ion homeostasis but also water balance. In our experiment 300 Zn caused significant decrease in water content both in the roots and shoots. The similar Zn dose (5 mM) also induced relative water content limitation in *R. sativus* plants (Ramakrishna and Rao 2014). The decline of water content in *B. vulgaris* plants was observed already at the concentration of 50 μM Zn, however this species seems to be Zn-sensitive because at this concentration all tested growth parameters decreased (Sagardoy et al. 2009).

The effect of Zn excess on plant growth parameters and morphology has been widely studied (Richard et al. 2011; Yang et al. 2011; Caldelas et al. 2012; Mangal et al. 2013; Mukhopadhyay et al. 2013; Ramakrishna and Rao 2014), however the research at the ultrastructural level has been scarce (Azzarello et al. 2012; Reboredo 2012; Xu et al. 2013; Liu et al. 2014).

The most evident ultrastructural alterations of root meristematic cells of *T. aestivum* plants treated with both tested Zn doses included increased vacuolization and accumulation of granular electron-dense deposits inside vacuoles and thickened cell walls. Additionally in the material treated with 300 Zn small electron-dense deposits were present in a thickened cell wall. Similar electron-dense material in the form of clumps or globules was observed in vacuoles of root cortical cells of *P. tomentosa* grown at 1000 μM Zn and these structures were identified in a confocal microscope using a specific Zn indicator (Azzarello et al. 2012).

It is known that accumulation of heavy metals in vacuoles and cell walls is the strategy to avoid metal induced toxicity (Frey et al. 2000). In *Thlaspi caerulescens* (Frey et al. 2000) and in *Potentilla griffithi* (Hu et al. 2009) Zn was accumulated in cell walls of root cortical cells. Zn presence in this compartment is usually accompanied with thickening of a cell wall (Jin et al. 2008; Liu et al. 2014), which together with increased cation exchange capacity, acts as an external barrier limiting Zn entry into a protoplast (Muschitz et al. 2009). It was also proved that proteins as well as cellulose, hemi-cellulose and pectins were Zn-binding components of plant cell walls (Sarret et al. 2009; Reboredo 2012). Zn localization mainly in vacuoles was observed in *Sedum alfredi* root cells (Jin et al. 2008). In vacuoles, Zn is usually bound to organic acids, mainly citrate that strongly binds metal ions (Frey et al. 2000; Broadley et al. 2007). The excess of metal is therefore safely stored there which prevents it from disturbing important metabolic processes in cytoplasm and organelles. In *T. aestivum* treated with 50 Zn there were only adaptive changes of root meristem cell structure. More numerous Golgi apparatus vesicles and thicker wavy cell wall suggested intensified process of cell wall component synthesis aiming at increasing the capacity of apoplast to sequestrate metal excess. Increased number of vacuoles filled with deposits indicated metal trapping. The cell ultrastructure did not show any degenerative changes which suggested that these mechanisms were effective. The only organelles significantly different from the control were mitochondria. The condensed form of mitochondria occurs when ADP level exceeds that of ATP for example due to inhibited oxidative phosphorylation. Such change was also induced by zinc excess in *P. tomentosa* (Azzarello et al. 2012). This type of structural alternations might be also trigged by imbalance of Fe and Cu ions observed in Zn-treated *T. aestivum* that could affect functioning of respiratory electron transport chain (Palmer and Guerinot 2009).

The nucleolar vacuoles which were present in 300 Zn treated *T. aestivum* cells suggested enhanced transcriptional activity in them. They are typical of plants in which the production of ribosomal ribonucleoproteins is lower than their migration into cytoplasm (Stępiński 2014). Vacuolated nuclei were also observed in root cells of Zn-treated *Nigella sativa* (El-Ghamery et al. 2003).

The first symptoms of wheat root cell degeneration processes *i.e.* thinned down cytoplasm with lipid droplets appeared at 300 Zn which might suggest that sequestration mechanisms were insufficient. Excess of Zn disturbs cell membrane integrity (Tsonev and Lidon 2012). In non-hyperaccumulating *Sedum alfredi* ecotype disruption of plasma membranes and severe plasmolysis were observed at 100 μM Zn^2+^ (Jin et al. 2008). Membrane injury estimated by the level of malondialdehyde (MDA) was noticed after Zn treatment in *R. sativus* (Ramakrishna and Rao 2014), *Camellia sinesis* (Mukhopadhyay et al. 2013) and *Hydrilla verticillata* (Xu et al. 2013).

In mesophyll cells of *T. aestivum* plants treated with both tested Zn concentrations no electron dense deposits indicating Zn presence were observed in TEM, while in mesophyll cells of *P. tomentosa* such deposits were seen in vacuoles (Azzarello et al. 2012). On the other hand, Zn stored in vacuoles of leaf epidermal cells of *T. cerulescens* was evenly distributed and no Zn containing crystals or deposits were observed (Frey et al. 2000). In our research there were no visible Zn deposits in vacuoles of leaf mesophyll cells of *T. aestivum*, while high concentration of this ion was measured in above-ground parts of plants, however since Zn content analyses were made in whole *T. aestivum* shoots the precise place of Zn accumulation cannot be specified. In *Saccharum* ssp. treated with Zn at the concentrations ranging from 0.065 to 130 mg L^−1^ the metal content in leaf lamina was constant while in stalk it increased (Jain et al. 2010). Moreover, significant difference in Zn concentrations even between leaf blade tissues may occur. High level of this ion was measured in epidermis but not in mesophyll cells of *T. cerulescens* (Küpper et al. 1999) while in *Arabidopsis halleri* the situation was opposite (Zhao et al. 2000).

At the ultrastructural level the toxic effects of Zn on wheat mesophyll cells were noticed only at the metal concentration of 300 mg L^−1^. The symptoms of plasmolysis correlated with decrease in water content were noticed in that experimental variant. The significant plasmolysis of mesophyll cells was also observed in *H. verticillata* treated with 150 μM Zn (Xu et al. 2013). The most evident effect of Zn consisted in changes of mitochondria and especially chloroplast shape resulting in protrusions. The proposed function of chloroplast protrusions is better compound exchange due to greater area of contact between plastids, mitochondria and microbodies and it seems to be one of the protective mechanisms to avoid oxidative stress (Holzinger et al. 2007).

Zn-triggered changes of chloroplast shape from ellipsoid to oval were visible in *P. tomentosa* (Azzarello et al. 2012) and *S. alfredii* (Jin et al. 2008) leaves together with starch and plastoglobule accumulation. In our experiment only increased number and size of starch grains were noticed. It suggests that in *T. aestivum* sugar metabolism was affected by the high Zn dose.

In other plant species the degenerative changes of mesophyll cell ultrastructure were observed at much lower Zn concentrations. In *Camelia sinensis* plants treated with 30 μM Zn chloroplasts had severely disorganised thylakoid system and swollen mitochondria (Mukhopadhyay et al. 2013). In 75-μM Zn-treated *H. verticillata* chloroplast, mitochondria and a nucleus were seriously altered (Xu et al. 2013). Comparing the above results with those for *T. aestivum* one can see that wheat is rather tolerant to Zn.

## Conclusions

The results of our experiment indicate that 50 Zn had no negative effects on *T. aestivum* while 300 Zn was suppressive to plant growth. Although wheat treated with 50 Zn took up relatively high amount of metal, no negative effects of that ion were noticed at the morphological and cytological levels. The probable results from the efficient adaptive modification, of root cell ultrastructure *i.e*. metal compartmentation in enlarged vacuoles. On the other hand, in spite of intensified metal sequestration in root cell vacuoles and even in cell walls in the plants treated with 300 Zn the negative effects of the metal on plant growth were visible; however, there were no severe symptoms of cell degeneration. The depletion of growth parameters might result not only from direct toxic effect of the tested metal but also from energy consuming defensive processes and disturbed microelement homeostasis as well as decrease in water content.
